# Assessing the power of principal components and wright’s fixation index analyzes applied to reveal the genome-wide genetic differences between herds of Holstein cows

**DOI:** 10.1186/s12863-020-00848-0

**Published:** 2020-04-28

**Authors:** M. G. Smaragdov, A. A. Kudinov

**Affiliations:** 1 Russian Research Institute of Farm Animal Genetics and Breeding - Branch of the l.K. Ernst Federal Science Center for Animal Husbandry, St. Petersburg, Pushkin Russia; 2 St. Petersburg, Russian Federation; 3 Department of Agricultural Science, University of Helsinki, FI-00014 Helsinki, Finland

**Keywords:** Principal components, Fixation index, Minor allele frequency, Dairy cattle, Genetic diversity

## Abstract

**Background:**

Due to the advent of SNP array technology, a genome-wide analysis of genetic differences between populations and breeds has become possible at a previously unattainable level. The Wright’s fixation index (F_st_) and the principal component analysis (PCA) are widely used methods in animal genetics studies. In paper we compared the power of these methods, their complementing each other and which of them is the most powerful.

**Results:**

Comparative analysis of the power Principal Components Analysis (PCA) and F_st_ were carried out to reveal genetic differences between herds of Holsteinized cows. Totally, 803 BovineSNP50 genotypes of cows from 13 herds were used in current study. Obtained F_st_ values were in the range of 0.002–0.012 (mean 0.0049) while for rare SNPs with MAF 0.0001–0.005 they were even smaller in the range of 0.001–0.01 (mean 0.0027). Genetic relatedness of the cows in the herds was the cause of such small F_st_ values. The contribution of rare alleles with MAF 0.0001–0.01 to the F_st_ values was much less than common alleles and this effect depends on linkage disequilibrium (LD). Despite of substantial change in the MAF spectrum and the number of SNPs we observed small effect size of LD - based pruning on F_st_ data. PCA analysis confirmed the mutual admixture and small genetic difference between herds. Moreover, PCA analysis of the herds based on the visualization the results of a single eigenvector cannot be used to significantly differentiate herds. Only summed eigenvectors should be used to realize full power of PCA to differentiate small between herds genetic difference. Finally, we presented evidences that the significance of F_st_ data far exceeds the significance of PCA data when these methods are used to reveal genetic differences between herds.

**Conclusions:**

LD - based pruning had a small effect on findings of F_st_ and PCA analyzes. Therefore, for weakly structured populations the LD - based pruning is not effective. In addition, our results show that the significance of genetic differences between herds obtained by F_st_ analysis exceeds the values of PCA. Proposed, to differentiate herds or low structured populations we recommend primarily using the F_st_ approach and only then PCA.

## Background

Farmed animals should have large genetic variation in exterior, production and fitness traits. Genetic variation is the basis for survival and maintaining of cattle populations. On genome level variation appears as considerable allelic diversity and heterozygosity. Genomic data help to track herds’ genetic divergence at molecular level. Knowledge of genetic diversity is also important for small breed conservation and crossbreeding strategies [1, 2]. Contemporary technologies allow to use massive SNPs data for these goals.

Several tools could be used to estimate genetic diversity in populations. The most effective and commonly used are principal component (PCA) and Wright’s fixation index (F_st_) analysis. Both methods are widely used to estimate genetic difference between populations. Genomic PCA finds the eigenvectors of the covariance matrix derived from the genotypes of animals. These eigenvectors provide the efficient linear combination of marker data which the most effectively differentiate of various samples, without requiring apriori sample classification information. The resolution of highly structured populations with PCA depends on non-random patterns of genetic variation. To reduce the impact of this factor, one should filter the data by removing a marker from every pair of markers which are in tight LD [3–5], or implement a shrinkage PCA [6], and apply iterative pruning PCA [7, 8].

More than 70 years have passed since S. Wright introduced a fixation index to measure genetic difference between populations [9]. His approach proved to be very fruitful for the further development of population genetics. Over these years many F_st_ statistics have been proposed. Among them the most commonly used estimators are those presented by Weir & Cockerham [10] and Nei [11]. But, the first one is sensitive to sample size and the second one consistently overestimates F_st_ [12]. Another approach used in the Hudson’s estimator [13]. It is not sensitive to sample size ratio, not systematically overestimate F_st_, and it is accurate and stable under various ascertainment schemes [12].

Generally accepted that the rare alleles play an important role in evolution. Analysis performed by Gorlov et al. [14] suggests that including rare SNPs in genotyping platforms will advance identification of causal SNPs in case-control association studies, particularly as sample sizes increase. This effect is confirmed by the genomic breeding value evaluation of dairy cattle [15] and the effect of rare alleles on estimated genomic relationships from whole genome sequence data [16]. For animals the frequency of SNPs alleles in the range from 0 to 0.5 obtained with arrays is nearly uniform while for sequence data this distribution is substantially biased to rare alleles [5]. Obtained phenomenon can lead to ascertainment bias in the evaluation between populations difference with SNPs arrays. Removing low-frequency and rare SNPs alleles (MAF < 0.02) can significantly distort results of PCA analysis [17]. Human population studies have shown inflation of ascertainment schemes on F_st_ values calculation [18–20]. In other studies were observed upward bias in F_st_ values [5, 21]. Clark et al. [22] on human populations demonstrated that data sets based on different ascertainment schemes gave different patterns of F_st_ values. Moreover, the raw array data and those with polymorphic SNPs in the wild chicken samples underestimated pairwise F_st_ values between breeds which had low F_st_ (< 0.15) in the whole genome resequencing (WGS) data, and overestimated this parameter for high WGS F_st_ (> 0.15) [5]. It should be borne in mind that F_st_ value can depend heavily on the level of variation present in a sample and the frequency of the most frequent allele [23]. Indeed, Jost [24] argued that F_st_ may be so affected by genetic diversity that it should not be used as a measure of population differentiation, gene flow or relatedness.

The Leningrad region is the highest average milk yield producing region in Russia, with approximately 60,000 cows of Holsteinized Black and White cattle. Dutch, Danish, and Swedish Black and White bulls and heifers were imported to Russia during the 1930s. The Black and White breed was officially registered in Russia in 1959. To improve milk traits of Black and White cattle, local farmers started to use imported from USA (since 1978) and the Netherlands (since 2002) Holstein bulls and semen. Currently, the commercial Russian Black and White cattle population can be considered as Holstein due to long-term crossing only with Holstein bulls.

In presented study, we tried to evaluate the following objective. 1. Evaluate the correspondence of MAF and linkage disequilibrium. 2. Assess the impact of outliers removal on F_st_ data. 3. Evaluate LD based pruning methodology on F_st_ values. 4. Evaluate impact the MAF of SNPs on F_st_ values. 5. Evaluate significance of F_st_ values. 6. Evaluate PCA analysis data. 7. Assess the power of F_st_ and PCA analyzes.

## Results

### Evaluation the correspondence of MAF and linkage disequilibrium

**Fig. 1:**
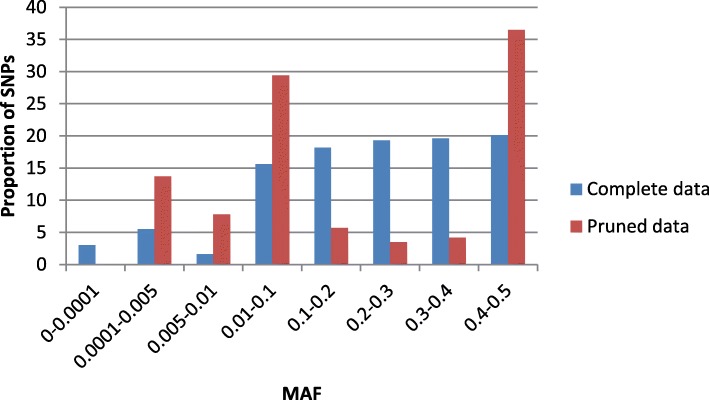
Proportion of SNPs in the complete and pruned data

The effect of LD - based pruning on the number of SNPs was large (see Additional file 1: Figure S1). To estimate impact of LD - based pruning on MAF of SNPs we calculated the distribution of MAF in eight bins (Fig. 1). The proportion of SNPs regarding the MAF bins in the complete and the pruned data was noticeably different. LD – based pruning completely removed monomorphic SNPs, disproportionally removed SNPs with MAF 0.2–0.4 while proportion of rare and common SNPs with MAF 0.0001–0.1 and 0.5 increased (Fig. 1). It can be suggested that in average SNPs with MAF 0.1–0.4 distributed in genome closer to each other than remaining SNPs leading to the largest LD between them.

### Assessing the impact of outliers removal on F_st_ data

On the first step we evaluated the impact of the outliers on F_st_ values. We calculated F_st_ values for Pairwise set of complete data both with correction and without correction on outliers (see Additional file 1: Table S1). After outliers correction in EIGENSOFT 799 cows and 46,626 SNPs were remained.

Absence of the outliers correction leads to a bias of F_st_ values but only for 6 from 78 pairs of the herds. In all cases the difference between F_st_ values was ±0.001 with exception of 4 and 13 pair of the herds having 0.002 difference. Nearly the same F_st_ values was also stored for Pairwise set where was excluded SNPs with MAF < 0.01. Among these F_st_ values only six pairs of the herds differed by 0.001 from F_st_ values for complete data in Table S2 (see Additional file 1) and three of them were the same as in result of outliers correction.

### Evaluation LD based pruning methodology on F_st_ values

Linkage disequilibrium pruning (LD < 0.1) had the same effect size on F_st_ values ±0.001 as the outliers had but affected more pairs of the herds 22 vs. 6 for outliers effect (Table 1 and see Additional file 1: Table 1). In point of fact the effect size on F_st_ was not large despite of considerable decrease in the total number of SNPs (5827 vs. 48,108) and their proportion in the SNPs bins (Fig. 1). Thus, LD - based pruning had a moderate effect on F_st_ values but it affected more pairs of herds.

**Table 1:** Estimates of F_st_ values for complete and pruned data

Herd	1	2	3	4	5	6	7	8	9	10	11	12	13
1		0.005^a^	0.005	0.006	0.006	0.004	0.006	0.004	0.003	0.005	0.005	0.005	0.004
2	**0.006**^**b**^		0.004	0.012	0.006	0.006	0.006	0.003	0.002	0.004	0.003	0.003	0.007
3	0.005	***0.003***		0.009	0.003	0.004	0.005	0.002	0.002	0.003	0.004	0.003	0.005
4	0.006	0.012	0.009		0.009	0.004	0.011	0.008	0.009	0.011	0.011	0.012	0.006
5	0.006	***0.005***	0.003	0.009		0.005	0.006	0.004	0.004	0.004	0.006	0.005	0.005
6	0.004	0.006	***0.003***	0.004	0.005		0.007	0.004	0.005	0.006	0.005	0.005	0.004
7	0.006	0.006	0.005	***0.010***	0.006	***0.006***		0.004	0.006	0.006	0.007	0.005	0.007
8	**0.005**	0.003	0.002	**0.009**	0.004	0.004	**0.005**		0.003	0.004	0.003	0.004	0.004
9	**0.004**	0.002	0.002	0.009	0.004	0.005	0.006	***0.002***		0.004	0.003	0.003	0.006
10	0.005	**0.005**	**0.004**	0.011	0.004	0.006	0.006	0.004	0.004		0.005	0.004	0.007
11	**0.006**	0.003	0.004	0.011	0.006	0.005	0.007	0.003	0.003	0.005		0.005	0.006
12	0.005	***0.002***	0.003	***0.011***	***0.004***	0.005	0.005	***0.003***	0.003	0.004	***0.004***		0.008
13	**0.005**	0.007	0.005	0.006	0.005	0.004	0.007	0.004	***0.005***	0.007	0.006	***0.007***	

^a^ F_st_ values for complete data are above the diagonal and F_st_ values for pruned data are below the diagonal

^b^ Increased F_st_ values for pruned data compared to complete data are in bold and decreased F_st_ values are in bold Italic. F_st_ values in bold are significantly different from those values in the complete data in range of *P* = 0.05–0.006 exept of the herds pair 4 and 12 which was insignificant)

### Evaluation impact of SNPs MAF on F_st_ values

**Fig. 2:**
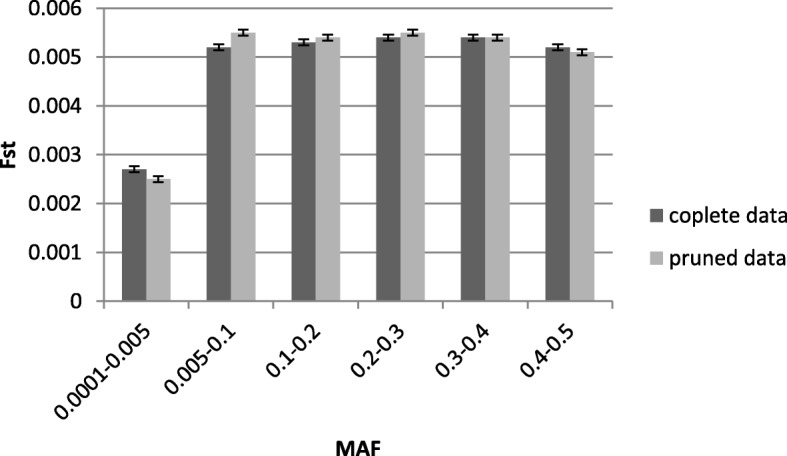
Dependence of mean F_st_ on the MAF range

To evaluate impact of SNPs MAF on F_st_ values, we divided the entire MAF interval 0.0001–0.5 into 6 bins and calculated for each of them the mean F_st_ value across Pairwise sets formed from complete and pruned data (Fig. 2). The rare SNPs alleles with MAF 0.0001–0.005 had the smallest mean F_st_ value (0.0027) across all herds than those for remaining SNPs (see Additional file 1: Table S3). It can be concluded that in average between herds differences calculated for rare alleles were less of those for common alleles. For MAF in the range of 0.1–0.5 the difference between the mean F_st_ values across beans for two Data sets was not significant. As a result of mutual compensation of the mean F_st_ values in complete and pruned data in whole MAF range, the total summed value of F_st_ value between them was insignificant (see Additional file 1: Table S3). Thus, these results again confirm a small effect size of LD – based pruning on F_st_ values only for rare SNPs not common SNPs alleles.

### Evaluation significance of F_st_ values

To assess significance of F_st_ values in the Table 1 we carried out the pairwise herds permutations of the cows treating them as H null-distribution. The results of these F_st_ values calculations are listed in Table S4 (see Additional file 1). Then, we calculate *P*-values for each pair of the herds in Pairwise set using Student’s t-test (Table 2). All of them were with P – values in the range from 1.0e-06 to 3.6e-60 with mean 6.5e-18 and median 3.6e-40, thereby it is higly skewed distribution. To calculate F_st_ for H null-distribution we carried out only 5 permutations for each of 78 pairs of the herds as it was time consuming process and result of P – values estimates would be only slightly underestimated. In the Table 1 the minimum F_st_ values were 0.002–0.003. The pairs of the herds corresponding to these values are the candidates for genetically most similar herds. However, when comparing these herds in Table 1 the errors were not taken into account. The probabilities of making a type 1 error for all 78 herd combinations are given in Table 2. To evaluate the genetic differences between the herds we have chosen cut off *P* ≤ 1.0e-30 (*P* ≤ 1.28e-32 taking into account the Bonferroni correction) in which, as a rule, the differences between the herds at F_st_ values 0.002–0.003 should be insignificant. The results are shown in the Table 3. Insignificant pairs of herds were 2 and 8, 9, 11, 12 (4 pairs); 3 and 5, 8, 9, 10 (4 pairs); 8 and 2, 3, 9, 11 (4 pairs); 9 and 2, 3, 11, 12 (4 pairs). The pairs of herds with 2, 3, 8 and 9 herds had 4–6 F_st_ values 0.002–0.003 (Table 1). Therefore, the results of identifying insignificant pairs of herds (Table 3) correspond to the minimal F_st_ data in the Table 1. In the Table 3 most significant pairs of herds at this cut off were the herd 4 (10 pars), 7 (12 pairs), 13 (12 pairs) or a more stringent level of significance at cut off P ≤ 1.28e-39 the herds 4, 7 and 13 had 10, 8 and 11 significant pairs of the herds (Table 3).

**Table 2:** Estimates between herds genetic differences (*P* – values) ^a^

Herd_Herd	PC 1	PC 1 Pruned data	PC 3	Summed 10 eigenvectors	Summed 20 eigenvectors	Summed 100 eigenvectors	Summed 100 eigenvectors Pruned dat	F_st_*P*-values
1_2	0.0031	0.0034	1.6E-05	4.2e-07	1.5e-11	3.0e-15	1.e-16	4.1e-49
1_3	0.0444	0.0460	0.0017	0.0002	1.3e-09	9.8e-18	4.1e-19	3.3e-47
1_4	0.0029	0.0029	0.0598	5.6e-05	4.6e-07	2.8e-10	4.7e-08	3.54e-32
1_5	0.8517	0.8652	0.0007	0.0289	6.6e-11	7.2e-17	5.0e-21	3.4e-40
1_6	0.6751	0.6748	0.6948	0.0504	7.81e-05	1.0e-13	6.4e-11	1.35e-38
1_7	0.0190	0.0202	0.1230	2.95e-07	3.1e-10	2.4e-25	1.4e-22	1.8e-54
1_8	0.0370	0.0381	0.0551	0.0137	9.5e-06	4.2e-08	2.5e-12	4.99e-37
1_9	0.2776	0.2854	0.0002	0.0056	0.0001	1.2e-10	5.4e-17	2.98e-26
1_10	0.0141	0.0146	0.0856	0.1030	0.0001	1.2e-12	1.1e-15	3.7e-35
1_11	0.0012	0.0013	0.0019	5.23e-05	7.4e-08	7.7e-14	3.0e-14	1.5e-45
1_12	0.0075	0.0079	0.0053	1.085e-5	1.9e-10	3.4e-18	7.0e-19	3.2e-34
1_13	0.4578	0.4554	0.6325	0.0141	0.0001	4.7e-19	1.0e-14	2.5e-40
2_3	0.1539	0.1561	0.0798	3.34e-06	6.7e-06	2.0e-14	1.4e-14	7.7e-42
2_4	1.0e-09	1.3e-09	1.8e-08	6.52e-17	9.9e-21	3.3e-24	6.0e-22	1.2e-54
2_5	0.0033	0.0034	0.7395	0.0001	1.6e-06	3.3e-18	1.2e-25	5.2e-59
2_6	0.0007	0.0007	6.1E-06	8.58e-7	8.2e-10	2.2e-16	3.8e-17	4.0e-58
2_7	0.2893	0.2868	0.0002	1.48e-14	1.9e-17	8.9e-26	4.7e-26	3.6e-60
2_8	0.4154	0.4179	0.0194	0.0002	8.9e-06	2.3e-11	2.5e-12	3.9e-28
2_9	0.0189	0.0194	0.7035	0.0156	0.0198	2.7e-11	5.1e-13	3.2e-20
2_10	0.8281	0.8312	0.0149	0.0119	0.0063	2.2e-12	1.7e-19	2.3e-41
2_11	0.6182	0.6189	0.1155	0.0020	1.7e-05	5.7e-06	4.7e-08	2.4e-27
2_12	0.8521	0.8502	0.1206	0.0003	0.00074	6.9e-11	1.1e-11	7.3e-20
2_13	2.9e-05	3.3e-05	0.0001	6.82e-15	1.6e-18	3.6e-26	7.7e-30	6.1e-49
3_4	1.0e-08	1.21e-08	2.4e-06	6.19e-13	1.80e-16	1.5e-21	1.2e-16	1.7e-51
3_5	0.0486	0.0484	0.2529	0.0063	0.0019	2.9e-13	7.1E-14	2.6e-26
3_6	0.0109	0.0115	0.0006	0.0004	6.4e-06	5.0e-12	7.9E-09	3.6e-40
3_7	0.6314	0.6428	0.0235	2.31e-05	3.7e-10	4.6e-22	8.9e-20	9.7e-48
3_8	0.6296	0.6306	0.3359	0.0615	0.0036	9.6e-06	2.0e-4	2.7e-19
3_9	0.2662	0.2661	0.2269	0.0089	0.01045	4.7e-10	4.3e-09	5.1e-16
3_10	0.2472	0.2479	0.2537	0.0052	0.00216	5.6e-10	9.6e-13	2.2e-25
3_11	0.0469	0.0475	0.8965	0.0088	2.9e-05	5.6e-10	3.7e-14	2.3e-39
3_12	0.2241	0.2272	0.9923	8.49e-06	8.9e-08	1.5e-12	1.9e-10	1.6e-19
3_13	0.0009	0.0009	0.0092	1.91e-05	3.6e-07	3.4e-23	1.5e-18	6.1e-49
4_5	0.0006	0.0007	5.0e-06	2.48e-07	2.8e-11	8.2e-17	2.5e-16	1.7e-51
4_6	0.0099	0.0100	0.1422	0.0949	0.1570	2.8e-12	2.5e-06	4.6e-23
4_7	4.4e-10	5.4e-10	0.0006	4.86e-14	9.7e-16	1.4e-30	1.5e-24	5.5e-45
4_8	1.37e-7	1.5e-07	0.0005	4.08e-09	1.0e-11	3.5e-15	8.4e-12	4.8e-46
4_9	3.0e-06	3.5e-06	5.2e-07	1.73e-11	2.9e-15	5.0e-24	3.3e-20	2.0e-44
4_10	2.8e-08	3.1e-08	0.0014	6.38e-09	1.7e-10	1.1e-19	6.0e-16	1.2e-41
4_11	2.9e-10	3.3e-10	4.7e-06	5.80e-16	2.1e-14	5.5e-18	7.7e-15	7.8e-50
4_12	5.9e-09	6.9e-09	2.2e-05	4.39e-11	4.1e-16	4.8e-26	1.6e-20	6.1e-53
4_13	0.0058	0.0061	0.0209	5.90e-06	3.2e-05	1.6e-22	2.1e-15	1.1e-39
5_6	0.5239	0.5355	0.0003	0.0013	1.1e-05	5.1e-15	9.1e-15	1.4e-33
5_7	0.0156	0.0160	0.0045	2.77e-06	4.4e-09	4.1e-24	3.3e-28	1.1e-40
5_8	0.0371	0.0371	0.0891	0.0008	0.0001	2.4e-11	7.2e-14	3.9e-37
5_9	0.3430	0.3429	0.9873	0.0755	5.5e-06	2.8e-15	1.9e-20	2.3e-42
5_10	0.0108	0.0109	0.0722	0.1244	0.02997	3.3e-13	6.4e-18	3.7e-36
5_11	0.0009	0.0009	0.3148	2.33e-07	1.3e-11	3.7e-14	4.9E-21	5.6e-56
5_12	0.0069	0.0070	0.3121	0.0024	5.9e-05	7.4e-17	3.2e-17	3.6e-40
5_13	0.3061	0.3144	0.0027	8.21e-05	4.4e-07	4.7e-20	5.3e-20	1.3e-54
6_7	0.0036	0.0038	0.0538	1.88e-08	9.8e-11	1.1e-27	9.4e-22	4.1e-43
6_8	0.0111	0.0114	0.0267	0.0020	2.8e-05	2.1e-12	2.1e-09	8.7e-35
6_9	0.1111	0.1154	8.6e-05	0.0010	7.3e-07	4.4e-17	1.0e-17	2.1e-47
6_10	0.0037	0.0038	0.0447	0.0015	0.0005	6.5e-17	3.2e-16	8.7e-39
6_11	0.0002	0.0002	0.0007	2.85e-06	2.5e-05	2.4e-12	3.9e-12	5.6e-45
6_12	0.0018	0.0019	0.0022	0.0001	2.7e-06	1.8e-17	2.9e-13	6.5e-34
6_13	0.7950	0.7922	0.3939	0.0002	0.00089	3.6e-14	1.8e-12	5.2e-40
7_8	0.9093	0.8995	0.4063	0.0002	1.9e-07	1.5e-15	1.0e-16	1.7e-38
7_9	0.1137	0.1171	0.0027	2.58e-09	3.9e-14	2.1e-27	1.2E-32	1.1e-57
7_10	0.3626	0.3556	0.4961	0.0005	3.0e-06	1.9e-25	4.5e-23	3.3e-37
7_11	0.0869	0.0854	0.0177	7.31e-12	1.5e-17	3.5e-24	5.2e-24	9.9e-46
7_12	0.3751	0.3723	0.0564	2.10e-05	4.6e-07	5.6e-25	2.2e-22	7.7e-33
7_13	0.0001	0.0001	0.3316	4.05e-13	2.2e-14	1.3e-33	4.4e-23	7.4e-47
8_9	0.1610	0.1612	0.0680	0.0061	0.0007	1.4e-06	2.0e-06	2.6e-26
8_10	0.5629	0.5626	0.8878	0.1035	0.0038	8.2e-12	7.7e-14	6.1e-34
8_11	0.1946	0.1956	0.3000	0.0718	0.0011	3.2e-07	4.4E-11	1.4e-25
8_12	0.5285	0.5321	0.4049	5.78e-06	2.2e-07	5.5e-10	5.6E-11	1.5e-33
8_13	0.0011	0.0011	0.1463	0.0061	9.3e-05	1.3e-14	6.2E-14	4.2e-42
9_10	0.0490	0.0493	0.0562	0.0362	0.0027	6.1e-13	2.1E-17	5.2e-40
9_11	0.0049	0.0050	0.2908	0.0234	0.0001	3.0e-09	6.0E-13	1.2e-30
9_12	0.0356	0.0363	0.2817	0.0001	1.2e-06	4.2e-15	8.5E-18	2.9e-30
9_13	0.0301	0.0316	0.0011	1.54e-08	6.4e-11	3.0e-22	1.2E-27	8.6e-59
10_11	0.4825	0.4852	0.2214	0.0009	2.1e-05	3.1e-17	1.7E-20	1.5e-33
10_12	0.9646	0.9658	0.3312	0.0108	0.0331	2.3e-12	2.1E-20	6.7e-38
10_13	0.0002	0.0002	0.2061	5.82e-06	6.7e-07	4.6e-25	1.7E-25	4.9e-43
11_12	0.4991	0.4976	0.9038	1.02e-07	1.3e-11	3.4e-18	2.8E-15	7.6e-47
11_13	5.5e-06	6.0e-06	0.0096	1.98e-09	2.2e-10	6.0e-16	6.0E-15	1.8e-38
12_13	9.1e-05	9.9e-05	0.0207	2.12e-14	5.6e-18	8.9e-26	2.4E-22	3.5e-53

^a^ complete data except for special marks in column names

**Table 3:** Between herds genetic differences for complete data revealed by F_st_ analysis

Herd	1	2	3	4	5	6	7	8	9	10	11	12	13
1		+	+		+	+	+	+	+		+	+	+
2	+		+	+	+	+	+			+			+
3	+	+		+		+	+				+	+	+
4		+	+		+		+	+	+	+	+	+	+
5	+	+		+		+	+	+	+	+	+	+	+
6		+					+	+	+	+	+	+	+
7	+	+		+	+	+		+	+	+	+	+	+
8				+						+		+	+
9				+		+	+			+			+
10		+		+									+
11	+			+	+		+					+	+
12				+				+	+				+
13	+	+	+	+	+	+	+	+	+	+		+	

+ − above diogonal denote significant genetic difference between pair of the herds at cutoff P ≤ 1.28e-32 (*P* - value adjusted by the Bonferroni correction). Below diagonal denote significant genetic difference between pair of the herds at cutoff P ≤ 1.28e-39 (*P* - value adjusted by the Bonferroni correction)

It was necessary to determine the most significant pairs of herds. The most significant at cut off *P* ≥ 1.28e-50 pairs of the herds were 2 and 5, 6; 4 and 2, 3, 5, 12; 5 and 11; 7 and 1, 2, 9; 13 and 5, 9, 12 (Table 2). These pairs of the herds correspond to the most genetically different pairs of the herds, while F_st_ data errors were taken into account as well. Summarizing the results of P - values calculating we can assert about a high level of significance the F_st_ analysis.

### Evaluation PCA analysis data

The eigenvalues of 100 eigenvectors calculated from the covariance matrix of alleles from 803 cows monotonically decreased from 9.5 to zero. It proves that the structure of the covariance matrix was enough homogeneous. Overall P- values and percent of variance (in brakets) for ten eigenvectors calculated for complete and pruned data were 2.8e-15 (1.16), 0.20 (1.05), 3.9e-14 (1.02), 1.9e-08 (0.88), 9.7e-03 (0.76), 2.3e-03 (0.72), 8.2e-03 (0.71), 6.0e-09 (0.66), 4.9e-05 (0.62), 5.6e-04 (0.59) (1) and 3.3e-16 (0.84), 6.4e-06 (0.79), 2.0e-04 (0.76), 3.4eE-06 (0.70), 2.6e-05 (0.67), 3.2e-08 (0.58), 2.0e-03 (0.55), 4.0e-04 (0.54), 2.2e-07 (0.53), 3.0e-03 (0.51) (2) respectively, i.e. they were similar. However the overall P - value for the second eigenvector of pruned data has become significant (6.4e-06) and at the same time overall P - value for third eigenvector on many orders of magnitude decreased (3.9e-14 vs. 2.0e-04). Such was the effect of LD - based pruning on overall P - values. From the list of overall P – values should be clear what main significant “axes of variation” were. From the list of variances for each eigenvector (1) and (2) can be calculated the variances to be used after summing ten eigenvectors. It were 8.17% for complete data and 6.47% for pruned data. Whence, the more eigenvectors will be summed, the more value of variance will be used.

**Fig. 3:**
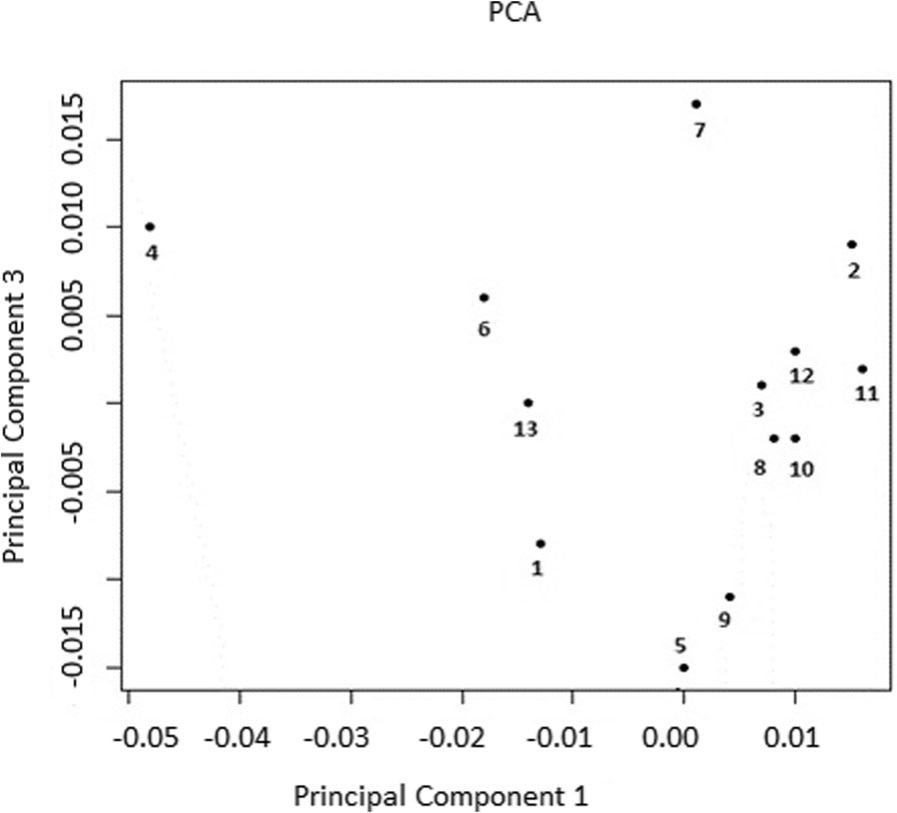
Position of mean F_st_ values of the herds along PC 1 and Pc 3. Each point denotes the mean herd position along PC 1 and PC 3 for complete data

**Fig. 4:**
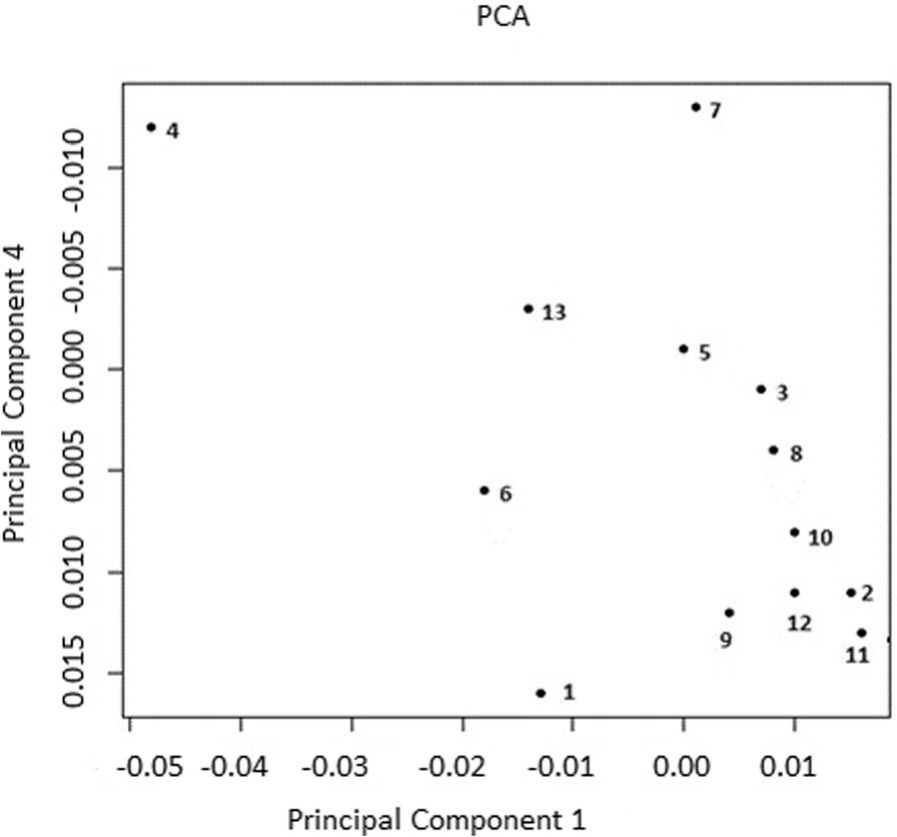
Position of mean F_st_ values of the herds along PC 1 and Pc 4. Each point denotes the mean herd position along PC 1 and PC 4 for complete data

Having the small F_st_ values and gradual decrease of the eigenvalues we calculated the mean for every herd in the PC scales to statistical description between herds genetic differences revealed by PCA. The plot of the means for all herds along PC 1 and PC 3 is shown on Fig. 3 and along PC 1 and PC 4 is shown on Fig. 4. To assess significance of genetic difference between 13 herds based on PC 1 we listed (+) (denoting between herds significance) in Table 4 obtained from P – values in Table 2 where cut off of significance was taken at *P* ≤ 0.05 but given the Bonferroni correction we get *P* ≤ 6.4e-4. Further, for brevity, we write P ≤ 0.05 instead P ≤ 6.4e-4. For PC 1 among 78 pairs of the herds there were 14 significant pairs of the herds. Most often significant data were observed for herds 4 and 13. Some significant results obtained with F_st_ statistic also confirmed with PCA for eigenvectors 1. For example, the greatest pairwise F_st_ – values for herd 4 were confirmed by noticeably higher level of significance revealed by PCA (Table 2). Furthermore, insignificant pairs of the herds 1 and 4, 4 and 6, 4 and 13 for PC 1 correspond to smallest F_st_ values for pairs of the herds formed with the herd 4 (Table 1). It should be noted a negligible effect size of LD based pruning on between herds’ significance for eigenvector 1 (Table 2).

**Table 4:** Between herds genetic difference for complete data revealed by PC 1 and PC 3

Herd	1	2	3	4	5	6	7	8	9	10	11	12	13
1													
2	+			+									+
3				+									
4		+	+		+		+	+	+	+	+	+	
5				+									
6		+	+		+								
7		+		+									+
8				+									
9	+			+		+							
10													+
11				+									+
12				+									+
13		+											

+ − denote significant genetic difference between pairs of the herds at cutoff P ≤ 0.05 (P - value adjusted by the Bonferroni correction is *P* ≤ 4.6e-4). The data for PC 1 are above diagonal and for PC 3 are below diagonal

The same procedure was carried out for PC 3 (Table 4). Among Pairwise set there were 16 significant pairs of herds. The most often significant data were obtained as well for herd 4 not herd 13. Out of fourteen significant pair of the herds revealed PC 1 only 9 coincide with sixteen significant pair of the herds revealed PC 3. Thus, PC 3 score is different from the PC 1 one. Obviously, it would be incorrect to make a conclusion about between herds significant differences if we used data for a separate eigenvector (Table 2).

Comparing the visible pattern of location the mean values of the herds along PC 1–3 and PC 1–4 we can draw some general conclusions (Figs. 3 and 4). The trajectory connecting herds 4–7–6-13-1 preserved on both figures. Other herds visually shifted relative to each other although not all of those displacements were significant at *P* < 0.05 as was shown along eigenvectors 1 and 3. However, the difference between these pairs of the herds was highly significant when we measured them with F_st_ statistics (Table 2). Thus, visual differences of the herds positions on Figs. 3 and 4 might be incorrect if we used only visual information along separate eigenvectors.

The lack of overall significance (*P* < 0.20) of second eigenvector for complete data and insignificance of the most pairs of the herds in Pairwise set indicates that there are not between herds genetic difference for this axis. Therefore, these data were excluded from consideration.

Furthermore, based on complete data, we tested the level of PCA data significance if P – values for Pairwise set were calculated from summed ten PC. Appropriate P – values are given in the Table 2 and significant pairs of the herds which were denoted as (+) are listed in the Table 5 at cutoff *P* ≤ 0.05. Among them there were 47 pairwise significant combinations of the herds for summed PC 1–10. The most significant result was obtained for herd 4 and 7 while insignificant results for herd 8, 9 and 10. Thus, giving summed genetic variance from 10 eigenvectors lead to noticeably increase the level of significance and change conclusions about data significance as was shown for PC 1 and PC 3.

**Table 5:** Between herds genetic difference for complete data revealed by summed PC 1–10 and PC 1–20

Herd	1	2	3	4	5	6	7	8	9	10	11	12	13
1		+	+	+			+				+	+	
2	+		+	+	+	+	+	+				+	+
3	+	+		+		+	+					+	+
4	+	+	+		+		+	+	+	+	+	+	+
5	+	+		+			+				+		+
6	+	+	+		+		+				+	+	+
7	+	+		+	+	+		+	+	+	+	+	+
8	+	+		+	+	+	+					+	
9	+			+		+	+					+	+
10	+			+	+		+		+				+
11	+	+	+	+	+	+	+		+			+	+
12	+		+	+	+	+	+	+	+		+		+
13	+	+	+	+	+		+	+	+	+	+	+	

+ − denote significant genetic difference between pair of the herds at cutoff P ≤ 0.05 (P - value adjusted by the Bonferroni correction is P ≤ 4.6e-4). The data for PC 1–10 are above diagonal and for PC 1–20 are below diagonal

To verify the level of significance further we calculated P – values for Pairwise set of the herds from complete data across summed 20 eigenvectors (Table 2). It turned out that for cutoff at P ≤ 0.05, 61 from 78 pairs of the herds were significant (Table 5). The most significant pairs of herds were 1, 4, 7, 8, 11 and 12 and the most insignificant pair of the herds was formed with the herd 3. Considering the data for summed ten and twenty eigenvectors, it is important to note that significant pairs of the herds varied greatly with an increase in the number of summed eigenvectors. Thus, increasing the number of summed eigenvectors leads to overall increase of significance level.

To include complete variance available from PCA analysis we calculated P – values for 100 summed eigenvectors (Table 2). For complete data *P*-values distribution had mean 2.2e-07 and median 2.2e-15, thereby the distribution is highly skewed. The herd 3 had minimum P - values with other herds (Table 2) therefore based on these values we selected significant pairs of the herds at cutoff *P* ≤ 1.0e-10 and given the Bonferroni correction *P* ≤ 1.28e-12. The results are shown in Table 6. The herd 3 formed 6 insignificant pairs of herds 3 and 6, 8, 9, 10, 11, 12 and herd 8 formed 9 insignificant pairs of the herds 8 and 1, 2, 3, 5, 6, 9, 10, 11, 12. Thus, the herd 8 and 3 was the most genetically related with other herds and this result do not contradict F_st_ values 0.002 and 0.003 prevailing in pairwise set for these herds (Table 1). It was necessary to determine the most significant pairs of the herds. The most significant pairs of the herds at cutoff P ≤ 1.28e-20 were 4 and 2, 3, 7, 9, 12, 13 (6 pairs); 7 and 1, 2, 3, 4, 5, 6, 9, 10, 11, 12, 13 (11pairs); 13 and 2, 3, 4, 7, 9, 10, 12 (7 pairs). This result for herds 7 and 13 is due to smaller standard errors for these herds than for herd 4 (see Additional file 1: Table 5).

**Table 6:** Between herds genetic differences for complete data revealed by PC 1–100

Herd	1	2	3	4	5	6	7	8	9	10	11	12	13
1		+	+		+	+	+			+	+	+	+
2			+	+	+	+	+			+			+
3				+	+		+						+
4		+	+		+		+	+	+	+	+	+	+
5							+		+	+	+	+	+
6							+		+	+		+	+
7	+	+	+	+	+	+		+	+	+	+	+	+
8													+
9				+		+	+			+		+	+
10							+				+		+
11							+					+	+
12				+			+						+
13		+	+	+			+		+	+		+	

+ − above diagonal denote significant genetic difference between pairs of the herds at cutoff P ≤ 1.28e-12 (*P* - value adjusted by the Bonferroni correction). Below diagonal denote significant genetic difference between pairs of the herds at cutoff P ≤ 1.28e-20 (*P* - value adjusted by the Bonferroni correction)

For pruned data P-values distribution had mean 2.6e-06 and median 1.8e-16. Thereby, the complete and pruned data distributions are similar. For the same cutoff P ≤ 1.28e-12 as for complete data, the data in the Table 2 were ranked (Table 7). The herd 3 formed 4 insignificant pairs of the herd 3 and 6, 8, 9, 12. The herd 8 formed 9 insignificant pair of the herd 8 and 1, 2, 3, 4, 5, 6, 9, 11, 12. Among 15 pair of herds 3 and 8 for complete data only 11 of those pairs coincide with pruned data. The most significant pair of the herds with cutoff P ≤ 1.28e-20 were 4 and 2, 3, 7 (3 pairs); 5 and 1, 2, 7, 11 (4 pairs); 7 and 1, 2, 4, 5, 6, 9, 10, 11, 12, 13 (10 pairs); 13 and 2, 7, 9, 10, 12 (5 pairs). Thus, P – values for complete and pruned data match good enough (except of the herds 4 and 5).

**Table 7:** Between herds genetic differences for pruned data revealed by summed PC 1–100

Herd	1	2	3	4	5	6	7	8	9	10	11	12	13
1		+	+		+		+		+	+	+	+	+
2			+	+	+	+	+		+	+			+
3				+	+		+			+	+		+
4		+	+		+		+		+	+	+	+	+
5	+	+					+		+	+	+	+	+
6							+		+	+		+	
7	+	+		+	+	+		+	+	+	+	+	+
8										+			+
9						+	+			+		+	+
10							+				+		+
11					+		+					+	+
12							+						+
13		+					+		+	+		+	

+ − above diagonal denote significant genetic difference between pair of the herds at cut off P ≤ 1.28e-12 (P ≤ 1.0e-10 was adjusted by the Bonferroni correction). Below diagonal denote significant genetic difference between pairs of the herds at cut off *P* ≤ 1.28e-20 (P ≤ 1.0e-18 was adjusted by the Bonferroni correction)

### Assessing the power of F_st_ and PCA analyzes

In the Table 2 listed P – values for Pairwise set of the herds calculated with PCA and F_st_ analyzes. According to these data for summed 100 eigenvectors, P – values were the smallest of those for any other eigenvector or summed 10 and 20 eigenvectors. This result was due to use the complete variance from initial data. Further, comparing P – values of PCA and F_st_ analyzes draw a conclusion that F_st_ P – values were many orders of magnitude less those of summed 100 eigenvectors. Across Pairwise set the PCA calculated power was within the range of 0.8–1.0, while for F_st_ it was within the range of 0.9–1 that is the probabilities of a type II error are similar. In total, considering by several orders of magnitude smaller P – values for F_st_, we can conclude that probability type I error for the F_st_ analysis was far less the PCA one. Therefore, it should be accepted that the data from the F_st_ analysis are more reliable.

## Discussion

Verification of the genetic diversity across herds is useful in a variety of biological context, especially in breeding, selection and conservation of breeds as well as crossbreeding strategies. In fact, the maintenance of genetic variation in the breeds is extremely important. The problem is to know whether such differentiation reflects meaningful differences. Genome – wide data allow to carry out the population analysis at unprecedented earlier level. We can expect herein to be able to resolve some diversity across herds’ genetic data. To solve this problem we apply two tools. First one was Wright’s F_st_ statistics [9]. Second one was recently proposed PCA tool as an alternative approach to determine within and between populations diversity [3].

### Comparing the power of F_st_ and PCA analyzes

Natural models of population structure predict that the most of the eigenvalues of theoretical covariance will be «small», nearly equal, and arise from sampling noise, while just a few eigenvalues will be «large», reflecting past demographic events [3]. It is not relevant for commercial dairy herds. Monotonic decrease of the eigenvalues was observed. This indicates a relatively homogeneous genetic structure of the herds due to a big enough gene flow between herds as result of artificial selection. For example the proportions of the cows born from the same bulls in 78 pair of the herds was up to 32% [25].

In fundamental analysis of genetic diversity with PCA, Patterson et al. [3] discovered a threshold (as measured, for example, by F_st_) below which the population structure was essentially undetectable. They proposed that for two equal size subpopulations, there is a threshold value of F_st_ calculated as 1/ √nm (where n is the number of animals and m is the number of SNPs), below which there will be essentially no evidence of the populations structure. In our study this threshold is within 0.0005–0.0006 for complete data which is considerably lower obtained minimal F_st_ value 0.002 and threshold 0.0015–0.0018 for pruned data which is comparable with minimal F_st_ value 0.002 (Table 1). Findings show that proximity to the threshold of PCA analysis for pruned data did not affect samples testing (Table 1).

Therefore, we predict that the power of our PCA analysis would be sufficient to reveal detectable across herds genetic differences. But, PCA calculates correctly if the markers are independent (between them have not sizable LD) [3, 17]. Several approaches have been proposed to achieve this goal, namely, shrinkage PCA [6], iterative pruning PCA [7, 8] and LD - based pruning [3]. We used LD - based pruning. For our local Holsteinized herds LD - based pruning has no affect on F_st_ and PCA data analysis. The same result for PCA was obtained when studying the genetic diversity of Spanish beef cattle at much greater F_st_ values (0.026–0.068) [4]. However, for human populations LD - based pruning has a sizable effect (e. g [3, 17].). Perhaps the PCA would be sensitive to LD - based pruning when populations or herds have pronounced genetic structure. It should be noted that after LD - based pruning the second eigenvector overall P - value became significant 6.4E-06 (see (2)). Consequently, insignificance of the second eigenvector may be possible result of LD between SNPs. Thus, the effect of LD on estimates between herds’ differences is moderate but not as great as for human population [3]. We propose this is a consequence of genetic relatedness of the cows in the herds. Really, the cows from 13 herds had considerable genetic relatedness owing to use high proportion up to 32% the same sires in the herds [25].

To clarify effect of LD on between herds differences, consider the results of F_st_ analysis. The pattern of MAF before and after LD - based pruning changes considerably (Fig. 1). Despite of this effect on MAF as pairwise F_st_ values (Table 1) and mean F_st_ values in MAF bins (see Additional file 1: Table S3) have not changed considarably. Meanwhile, the rare alleles have the smaller mean F_st_ values than common alleles, particularly for MAF 0.0001–0.005 (see Additional file 1: Table S3) and F_st_ values gradually increase up to MAF 0.1. Thus, the rare SNPs alleles are less differentiated between the herds than common alleles but they did not have substantial effect on F_st_ values. The less differentiation rare alleles may be suggested as a by-effect of artificial selection on the highest breeding values.

It was proposed if population has gone through bottleneck then F_st_ values could be greater for rare alleles as compared with common alleles [3]. We have observed the opposite effect, a decrease of F_st_ values for rare alleles. This means the bottleneck event in the breeding history of the herds if it had occurred it might have revealed by PCA.

The PCA plots of the herds means along first, third and fourth PC for the cows from complete data are shown on Figs. 3 and 4. The position of forth herd is outstanding on these images. The mean pairwise F_st_ value with other 12 herds for herd 4 is also the greatest 0.0087 compared with those of other 12 herds (0.0038–0.0063) [25]. We assume that this result is caused by the heavy use of bulls from the Netherlands between 2000 and 2007 years in herd 4, while bulls imported mainly from the USA and Canada were more recently used in the other 12 herds. Therefore, all pairwise combinations of the forth herd with other twelve herds are highly significant for first and third (except of pair the herds 1 and 4, 6 and 4), summed PC 1–10 and PC 1–20, summed 10–100 eigenvectors (except 4 and 6 pair of the herds) (Table 2).

Unlike the position of the herd 4, the position of third and eighth herds was nearly in the middle of the cluster which located herds 3, 8 10, 11, 12 on Fig. 3. These herds have minimal mean pairwise F_st_ – values with other 12 herds 0.0038 and 0.0039 [25]. The herd 3 forms many insignificant at *P* ≤ 0.05 pairwise herds combinations with other herds revealed by PCA 1–20 (Table 5) and at *P* ≤ 1.26e-12 by PC 1–100 (Table 6). Such properties of third herd are the result of genetic relatedness of the cows from these herd with the cows of other 12 herds mostly due to a large percentage of cows (up to 32%) born from the same bulls used in other 12 herds [25]. The same is true for herd 8 revealed by PC 1–100 (Table 6). The herd 13 is in the middle of both images on Figs. 3 and 4. Therefore, the herd 13 forms many highly significant pairs of herds revealed by PC 1–100 and F_st_ (Tables 2, 6 and 7).

Thus, mutual position of the herds and their pairwise significance depends on the eigenvectors since they are orthogonal and in each of them used only a part of genetic variance. It cannot be used the certain eigenvector to evaluate genetic differences for low genetically different pairs of the herds. Only summed eigenvectors are able to accurately assess these differences not contradictory to F_st_ approach. This conclusion is fully confirmed by the results obtained from summed 100 eigenvectors (Table 2).

### Examples between cattle breeds genetic differences

For the large – scale SNP data the PCA and F_st_ are widely used to summarize the structure of genetic variation in the populations. Consider some findings available from publications studying a moderate between populations difference. Analysis of Russian cattle breeds demonstrate a very low differentiation of Black and White breed from Holstein - Friesian breed (Fst = 0.02) [26]. The authors did not use PCA. In another research F_st_ value for Black and White and Holstein breeds was 0.035 and Black and White breed formed a cluster with the breeds from Northern Europe on multi dimentional scaling (MDS) images [27]. PCA analysis applied to a distance matrix based on identity by state (IBS) showed a grouping of Spanish beef cattle breeds [4]. The degree of genetic differentiation was small to moderate as the pairwise fixation index of genetic differentiation among breeds estimates ranged from 0.026 to 0.068. Obtained results indicate large within-breed diversity and a low degree of divergence among the autochthonous Spanish beef cattle breeds studied. Among 47 worldwide breeds the USA and French Holstein have F_st_ value 0.004 and they are indistinguishable across PC 1 – PC 2 [28]. Authors concluded that PCA may fail to detect spatial structuring if this is not associated with the most pronounced genetic differentiation. Some degree of differentiation was shown with PCA between the USA and New Zealand Jersey bulls and cows [29]. The mean (max) F_st_ across the genome for AU versus US cows was 0.008 (0.12) and the average (max) for US versus AU, US versus NZ, and AU versus NZ was 0.006 (0.08), 0.029 (0.21) and 0.009 (0.07), respectively. Authors suggest that although some differentiation based on F_st_ was seen, especially for US versus NZ cows, the other populations appear to be similar. Noteworthy, differentiation between Australian and the USA Jersey cow populations was marginal in comparison with populations of the bulls. On PC 1 – Pc 2 image it was impossible to differentiate them geographically. Taken together the PCA and F_st_ results show that two artificially unselected breeds were not well differentiated and still cover a considerable part of the original genetic diversity [30]. On the contrary, artificially selected breeds show significantly highest differentiation. The highest overlap of genetic variation was found between Anatolian Black and Illyrian Mountain Buša (F_st_ = 0.037). This breeds were very close to each other in the PC 1 - PC 2 and PC 1 - PC 3 images and statistical prove on genetic differences between them are not given. Most of the remaining breeds also had their smallest F_st_ value (F_st_ = 0.037–0.096) when compared to Illyrian Mountain Buša. In indigenous six cattle populations of Ethiopia and Korea, PCA evidently distinguishes Ethiopian cattle populations from Hanwoo breed [31]. The most similar populations are Ambo – Arsi, Horro wih F_st_ 0.002 (*P* < 0.01) and they are very close to each other on PC 1 – PC 2 image but statistical data are not shown. Ancestry analysis of the new world cattle evidences that the first axis of PC was associated with the indicine – taurine split and the second PC axis was associated with the divergence between European and African taurine cattle [32]. The authors calculated the overall *P*-values based on TracyWidom test and shown that 154 axes in the 50 k dataset were statistically significant. In another research PCA and F_st_ showed minimal structure within the Guernsey breed, with no complete segregation of animals reflecting geographic origin (Fst = 0.006–0.011) and PCA with no distinct visual animal separation [33].

It is important to note that as a rule in above mention publications the eigenvalues (variance) decrease faster than in our study. Apparently this is the result a considerably low between herds genetic difference comparing to the breeds. We should keep in mind that visual evaluations of the genetic distance between herds on PCA images may be incorrect without of statistical prove. As a rule, any statistical treatment of PCA between populations’ results was not given and such images are only illustrative.

What conclusions from obtained results should be done regarding the power of F_st_ and PCA analyzes? Wright’s F_st_ is based on maximization of allele frequencies differences between populations through variance component. Used by us Hudson’s estimator provides the genetic difference between populations compared to the genetic difference within populations through variance component as well. Applied in our research PCA relies on covariance matrix of SNPs alleles among animals [3] and it able to find between herds genetic variation. That is F_st_ and PCA based on similar mathematical approaches and additional simulation analysis is needed to determine why F_st_ analysis gives more significant data. Summarizing the results of F_st_ and PCA analyzes should be noted that the power of both analyzes similar but probability of making a type I error is much less for F_st_ approach. It can be concluded at that point the F_st_ analysis is superior to PCA.

## Conclusions

Firstly, despite of genetic relatedness of the cows in the herds, F_st_ and PCA analyzes are able to differentiate between herds genetic differences. But, PCA applied to the herds might only be efficient when summed results of several eigenvectors will be used. Secondly, despite of considerable change in the number of SNPs and their MAF spectrum due to LD - based pruning, it has a small effect on the results of F_st_ and PCA analyzes. We suggest that this is a consequence of homogeneous genetic structure of the herds. Our findings show that F_st_ method give the more significant data than PCA but PCA approach might be useful due to visualization of some genetics features of the herds.

## Methods

### Animal resources and SNP genotyping

Data and genotypes were obtained from Committee on Agro-Industrial Complex of Leningrad region. Cows genotypes were available from 13 breeding herds locating in Leningrad region (Russia) born in range from 2010 to 2013. Animals for genotyping were selected by farmers with respect to pedigree structure of the herd. Number of animals genotyped depends on number of sires used in herd at least one daughter was presented by sire. In case of multiple daughters were presented per single sire, sire of dams were different. Sampled animals are presented 8–15% of total number of milking cows (see Additional file 1: Table S5). Altogether, 500 cows were genotyped with BovineSNP50 v.2.0 array (Illumina Ca. USA) and 300 cows with BovineSNPIDBv3 array (Illumina Ca. USA). In the first quality control step, SNPs with quality score less than 0.7 were removed. Then, imputation of the BovineSNPIDBv3 chip data (40 K SNPs) to BovineSNP50 v.2.0 chip data (50 K SNPs) was carried out with the Beagle software [34]. Genotyping quality control (QC) was done with PLINK 1.9 [35]. Only autosomal chromosome were considered. Three Data sets (Ds) were done by stepwise adding of QC criteria. In complete Ds missing rate per SNP was no more than 5% and probability of deviation from Hardy-Weinberg equilibrium (HWE) was less than 1.0E-03. It includes 804 cows which were genotyped with 48,108 SNPs (Total genotyping rate was > 0.99). In other Ds SNPs with MAF < 0.01 were removed resulted in 43,298 SNPs [25]. To pruned data, LD (0.1) - based SNPs pruning with –indep command in PLINK was applied to obtain pruned data, including only 5827 SNPs. Further, for each sample 78 pairwise comparisons between 13 herds (hereafter called Pairwise set) were formed.

### F_st_ analysis

The F_st_ values were estimated with EIGENSOFT 6.0.1 software [3], where Hudson’s estimator was implemented. The standard errors of Hudson’s F_st_ estimates were calculated using a block-jackknife approach implemented in EIGENSOFT. To estimate significance of F_st_ values the permutations of the cows between each pair of the herds was carried out to get the distribution under H_0_ hypothesis. We used PLINK commands –make –pheno and –fiter –mfilter 5 to perform 5 pairwise permutations. Then within each of permuted pair of the herds, the cows were allocated into two groups with the same size as the original pairs of the herds had and they were coded as case and control. Further, we calculated F_st_ value for each of 5 permuted pairs of the herds. Finally the mean F_st_ value and standard error for each 5 permuted pair of the herds was calculated. Altogether, 78 mean F_st_ values under H_0_ distribution were calculated and then 78 P – values were estimated using one-sided Student’s t-test. Power of t-test was calculated with «powerAnalysis» program in R software environment [36]. The standard error of mean F_st_ for MAF in the range 0.0001–0.5 was calculated as MSE = $\frac{1}{m}\sqrt{\sum_1^m{SE}_i^2}$ where m is the number of evaluated MAF bins equal to 6. When calculating the MSE for F_st_ value in each bin, m was equal to 78 pairs of the herds.

### Principal components analysis

For calculation of PCA the EIGENSOFT 6.0.1 software was used [3]. The outliers removal was carried out during each iteration. We used 6 standard deviations which an animal must exceed along one of the top principal components in order to be removed as an outlier. ANOVA *P*-values between each pair of the herds along principal components were calculated. For each pair of the herds, the above mentioned ANOVA statistics are summed across 10, 20 up to 100 eigenvectors. The distributions were approximately chisq with d.f. equal to the number of eigenvectors minus one. Likewise P-values were calculated for each 78 pairs of herds including summed PC 1–10, PC 1–20 and PC 1–100. For each of leading component PC 1, PC 3 and PC 4 the mean herd value was calculated. Then, they were plotted with R software environment [36]. Power of PCA analysis for summed PC 1–100 eigenvectors was calculated with *«*powerAnalysis» software in R software environment [36]. When comparative evaluating P - values in Table 2 Bonferroni corrections by formula: P = α/m was used, where α is the desired overall alpha level and m is the number of hypotheses.

## Supplementary information

**Additional file 1: Table S1.** Effect of outliers on estimates of F_st_ values for complete data. ^a^ - F_st_ values for complete data corrected on the outliers are above the diagonal and F_st_ values for complete data does not corrected on the outliers are below the diagonal. ^b^ - Increased F_st_ values are in bold and decreased F_st_ values are in bold Italic. **Table S2.** Effect of rare alleles with MAF < 0.01 on estimates of F_st_ values. ^a^ - F_st_ values for complete data after removal of the alleles with MAF < 0.01 are below the diagonal and F_st_ values for complete data does not corrected on MAF < 0.01 are above the diagonal. ^b^ - increased F_st_ values are in bold and decreased F_st_ values are in bold Italic. **Table S3.** Mean F_st_ values across Pairwise set of the complete data in MAF bins. * - In each MAF bin 78 F_st_ values was averaged. Statistical estimates were obtained with t-test. ** - MSE calculation see at materials and methods. **Table S4.** Estimates of F_st_ values calculated for H_0_ distribution. F_st_ values should be multiplied by 10^− 4^. **Table S5.** Standard errors of the F_st_ – values computed by EIGENSOFT 6.0.1. Standard errors of F_st_ obtained from complete data are above diagonal and from pruned data are below diagonal. SE values should be multiplied by 10^− 4^. **Table S6.** Description of the herds and number of the genotyped cows. * - Country of origin of the sires of the genotyped cows, NL – the Netherlands. **Figure S1.** Effect of LD - based pruning on the number of SNP in the complete data.

## Data Availability

The cows genotypes used during the current study are available from the corresponding author upon a reasonable request.

## Abbreviations

F_st_: Wright’s fixation index
PCA: Principal Components Analysis
LD: Linkage Disequilibrium
