# Supplementary material for: Assessing the power of principal components and wright’s fixation index analyzes applied to reveal the genome-wide genetic differences between herds of Holstein cows
Source: BMC Genet. 2020 Apr 28;21:47. doi: 10.1186/s12863-020-00848-0 (PMC7189535; doi:10.1186/s12863-020-00848-0)

Additional material

**Table S1** Effect of the outliers on estimates of F_st_ values for complete data

| Herd | 1 | 2 | 3 | 4 | 5 | 6 | 7 | 8 | 9 | 10 | 11 | 12 | 13 |
| --- | --- | --- | --- | --- | --- | --- | --- | --- | --- | --- | --- | --- | --- |
| 1 |  | 0.005^a^ | 0.005 | 0.006 | 0.006 | 0.004 | 0.006 | 0.004 | 0.003 | 0.005 | 0.005 | 0.005 | 0.004 |
| 2 | **0.006^b^** |  | 0.004 | 0.012 | 0.006 | 0.006 | 0.006 | 0.003 | 0.002 | 0.004 | 0.003 | 0.003 | 0.007 |
| 3 | 0.005 | 0.004 |  | 0.009 | 0.003 | 0.004 | 0.005 | 0.002 | 0.002 | 0.003 | 0.004 | 0.003 | 0.005 |
| 4 | 0.006 | 0.012 | 0.009 |  | 0.009 | 0.004 | 0.011 | 0.008 | 0.009 | 0.011 | 0.011 | 0.012 | 0.006 |
| 5 | 0.006 | 0.006 | 0.003 | 0.009 |  | 0.005 | 0.006 | 0.004 | 0.004 | 0.004 | 0.006 | 0.005 | 0.005 |
| 6 | 0.004 | 0.006 | 0.004 | 0.004 | 0.005 |  | 0.007 | 0.004 | 0.005 | 0.006 | 0.005 | 0.005 | 0.004 |
| 7 | 0.006 | 0.006 | 0.005 | ***0.010*** | 0.006 | 0.007 |  | 0.004 | 0.006 | 0.006 | 0.007 | 0.005 | 0.007 |
| 8 | ***0.003*** | 0.003 | 0.002 | 0.008 | 0.004 | 0.004 | 0.004 |  | 0.003 | 0.004 | 0.003 | 0.004 | 0.004 |
| 9 | 0.003 | 0.002 | 0.002 | 0.009 | 0.004 | 0.005 | 0.006 | 0.003 |  | 0.004 | 0.003 | 0.003 | 0.006 |
| 10 | 0.005 | 0.004 | 0.003 | ***0.010*** | 0.004 | 0.006 | 0.006 | 0.004 | 0.004 |  | 0.005 | 0.004 | 0.007 |
| 11 | 0.005 | 0.003 | 0.004 | 0.011 | 0.006 | 0.005 | 0.007 | 0.003 | 0.003 | 0.005 |  | 0.005 | 0.006 |
| 12 | **0.006** | 0.003 | 0.003 | 0.012 | 0.005 | 0.005 | 0.005 | 0.004 | 0.003 | 0.004 | 0.005 |  | 0.008 |
| 13 | **0.005** | 0.007 | 0.005 | 0.006 | 0.005 | 0.004 | 0.007 | 0.004 | 0.006 | 0.007 | 0.006 | 0.008 |  |

^a^ - F_st_ values for complete data corrected on outliers are above the diagonal and F_st_ values for complete data does not corrected on outliers are below the diagonal.

^b^ - Increased F_st_ values are in bold and decreased F_st_ values are in bold Italic.

**Table S2** Effect of rare alleles with MAF < 0.01 on estimates of F_st_ values

| Herd | 1 | 2 | 3 | 4 | 5 | 6 | 7 | 8 | 9 | 10 | 11 | 12 | 13 |
| --- | --- | --- | --- | --- | --- | --- | --- | --- | --- | --- | --- | --- | --- |
| 1 |  | 0.005^a^ | 0.005 | 0.006 | 0.006 | 0.004 | 0.006 | 0.004 | 0.003 | 0.005 | 0.005 | 0.005 | 0.004 |
| 2 | 0.005 |  | 0.004 | 0.012 | 0.006 | 0.006 | 0.006 | 0.003 | 0.002 | 0.004 | 0.003 | 0.003 | 0.007 |
| 3 | 0.005 | 0.004 |  | 0.009 | 0.003 | 0.004 | 0.005 | 0.002 | 0.002 | 0.003 | 0.004 | 0.003 | 0.005 |
| 4 | ***0.005***^b^ | 0.012 | 0.009 |  | 0.009 | 0.004 | 0.011 | 0.008 | 0.009 | 0.011 | 0.011 | 0.012 | 0.006 |
| 5 | 0.006 | 0.006 | 0.003 | 0.009 |  | 0.005 | 0.006 | 0.004 | 0.004 | 0.004 | 0.006 | 0.005 | 0.005 |
| 6 | ***0.003*** | 0.006 | 0.004 | 0.004 | 0.005 |  | 0.007 | 0.004 | 0.005 | 0.006 | 0.005 | 0.005 | 0.004 |
| 7 | 0.006 | 0.006 | 0.005 | ***0.010*** | 0.006 | 0.007 |  | 0.004 | 0.006 | 0.006 | 0.007 | 0.005 | 0.007 |
| 8 | 0.004 | 0.003 | 0.002 | 0.008 | 0.004 | 0.004 | 0.004 |  | 0.003 | 0.004 | 0.003 | 0.004 | 0.004 |
| 9 | 0.003 | 0.002 | 0.002 | 0.009 | 0.004 | 0.005 | 0.006 | 0.003 |  | 0.004 | 0.003 | 0.004 | 0.006 |
| 10 | 0.005 | 0.004 | 0.003 | ***0.010*** | 0.004 | 0.006 | 0.006 | 0.004 | 0.004 |  | 0.005 | 0.004 | 0.007 |
| 11 | 0.005 | 0.003 | 0.004 | 0.011 | 0.006 | 0.005 | 0.007 | 0.003 | 0.003 | 0.005 |  | 0.005 | 0.006 |
| 12 | ***0.004*** | 0.003 | 0.003 | 0.012 | 0.005 | 0.005 | 0.005 | 0.004 | 0.003 | 0.004 | 0.005 |  | 0.008 |
| 13 | 0.004 | 0.007 | 0.005 | **0.008** | 0.005 | 0.004 | 0.007 | 0.004 | 0.006 | 0.007 | 0.006 | 0.008 |  |

^a^ - F_st_ values for complete data after removal of the alleles with MAF < 0.01 are below the diagonal and F_st_ values for complete data does not corrected on MAF < 0.01 are above the diagonal.

^b^ - increased F_st_ values are in bold and decreased F_st_ values are in bold Italic.

**Table S3** Mean F_st_ values across Pairwase set of complete data in MAF bins

| MAF* | Mean F_st_ (complete dataI) | MSE** | Mean F_st_ (pruned data) | MSE | P - value |
| --- | --- | --- | --- | --- | --- |
| 0.0001-0.005 | 0.0027 | 0.000043 | 0.0025 | 0.000064 | 0.01 |
| 0.005-0.1 | 0.0052 | 0.000034 | 0.0055 | 0.000069 | 0.0001 |
| 0.1-0.2 | 0.0053 | 0.000035 | 0.0054 | 0.00013 | 0.44 |
| 0.2-0.3 | 0.0054 | 0.000040 | 0.0055 | 0.000052 | 0.13 |
| 0.3-0.4 | 0.0054 | 0.000038 | 0.0054 | 0.000050 | 1.0 |
| 0.4-0.5 | 0.0052 | 0.000044 | 0.0051 | 0.000051 | 0.14 |
| 0.0001-0.5 | 0.00486 | 0.000016 | 0.00493 | 0.00005 | 0.19 |
| 0.005-0.01 | 0.0038 | 0.000074 | 0.0041 | 0.000094 | 0.01 |

* - In each MAF bin 78 F_st_ values was averaged. Statistical estimates were obtained with t-test.

** - MSE calculations see at materials and methods.

**Table S4** Estimates of F_st_ values calculated for H_0_ distribution

________________________________________________________________________________

Herd 1 2 3 4 5 6 7 8 9 10 11 12 13

1 2.0 2.0 -4.0 -4.0 2.0 -1.0 -2.0 2.0 -3.0 2.0 0.1 -2 0

2 1.0 0.0 2.0 2.0 1.0 1.0 -2.0 -2.0 2.0 -2.0 0.1

3 -1.0 2.0 -1.0 2.0 -2.0 -1.0 2.0 1.0 -2.0 2.0

4 -1.0 0.1 -1.0 0.1 -1.0 -2.0 0.1 0.1 -4.0

5 -3.0 -4.0 0.1 -2.0 -10 0.1 -2.0 2.0

6 1.0 2.0 2.0 -3.0 2.0 -3.0 -2.0

7 -2.0 2.0 -2.0 0.1 -1.0 -1.0

8 2.0 -3.0 2.0 -4.0 -2.0

9 -2.0 -1.0 -2.0 2.0

10 -4.0 -2.0 0.1

11 2.0 -2.0

12 -1.0

_______________________________________________________________________________________

F_st_ values should be multiplied by 10^-4^.

**Table S5** Standard errors of the F_st_ – values computed by EIGENSOFT 6.0.1

________________________________________________________________________________

Herd 1 2 3 4 5 6 7 8 9 10 11 12 13

1 2.0 2.0 3.0 3.0 2.0 3.0 2.0 2.0 3.0 2.0 3.0 2.0

2 3.0 2.0 4.0 2.0 2.0 2.0 2.0 2.0 2.0 2.0 2.0 3.0

3 2.6 2.4 3.0 2.0 2.0 2.0 2.0 2.0 2.0 2.0 2.0 2.0

4 4.0 5.0 4.5 3.0 3.0 4.0 3.0 4.0 4.0 4.0 4.0 3.0

5 3.0 3.4 3.0 4.4 3.0 3.0 2.0 2.0 2.0 2.0 2.0 2.0

6 3.6 3.0 2.8 4.9 3.4 3.0 2.0 2.0 3.0 2.0 3.0 2.0

7 2.6 2.7 2.5 3.7 3.0 3.3 2.0 2.0 3.0 3.0 3.0 3.0

8 2.6 2.3 2.9 3.5 2.7 3.0 2.5 2.0 2.0 2.0 2.0 2.0

9 2.7 2.7 2.4 3.8 2.4 2.2 2.4 2.5 2.0 2.0 2.0 2.0

10 3.7 2.8 4.7 5.1 3.7 3.6 2.9 3.5 2.7 3.0 2.0 3.0

11 2.7 2.9 2.4 4.5 2.8 2.8 2.7 3.0 2.7 3.0 2.0 3.0

12 2.8 2.8 2.9 5.0 3.0 3.4 3.0 3.3 2.4 3.0 3.0 3.0

13 3.0 3.4 2.7 4.4 2.5 3.1 2.8 2.2 2.5 3.4 3.0 3.4

_____________________________________________________________________________________

Standard errors of F_st_ obtained from complete data are above diagonal and from pruned data are below diagonal. SE should be multiplied by 10^-4^.

Table S6 Description of the herds and number of the genotyped cows

| Herd | Russia USA Canada NL* | The number of cows  in the herd | Sample of the cows | Milk yield  (305 days) |
| --- | --- | --- | --- | --- |
| 1 | 54 13 33 | 870 | 57 | 9590 |
| 2 | 3 69 15 13 | 1009 | 85 | 10795 |
| 3 | 13 56 9 22 | 985 | 73 | 9215 |
| 4 | 100 | 657 | 44 | 8664 |
| 5 | 16 21 21 42 | 537 | 58 | 9723 |
| 6 | 28 16 3 53 | 697 | 54 | 8247 |
| 7 | 38 31 24 7 | 698 | 57 | 8853 |
| 8 | 14 43 10 33 | 662 | 53 | 9287 |
| 9 | 67 20 13 | 911 | 76 | 10166 |
| 10 | 10 52 5 33 | 551 | 44 | 11325 |
| 11 | 7 70 4 19 | 502 | 63 | 8847 |
| 12 | 10 64 3 33 | 1013 | 62 | 8086 |
| 13 | 8 11 12 69 | 555 | 64 | 9277 |

* - Country of origin of the sires of the genotyped cows, NL – the Netherlands

**Figure S1** Effect of LD - based pruning on the number of SNPs in the complete data


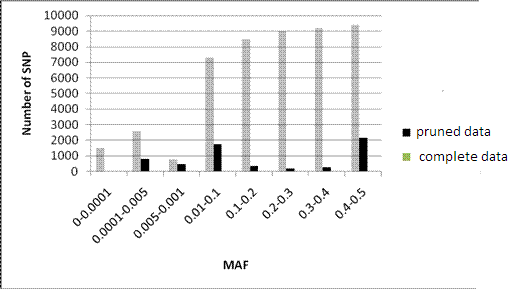

Supplement: Supplementary file 1 — Additional file 1: Table S1. Effect of outliers on estimates of Fst values for complete data. a - Fst values for complete data corrected on the outliers are above the diagonal and Fst values for complete data does not corrected on the outliers are below the diagonal. b - Increased Fst values are in bold and decreased Fst values are in bold Italic. Table S2. Effect of rare alleles with MAF < 0.01 on estimates of Fst values. a - Fst values for complete data after removal of the alleles with MAF < 0.01 are below the diagonal and Fst values for complete data does not corrected on MAF < 0.01 are above the diagonal. b - increased Fst values are in bold and decreased Fst values are in bold Italic. Table S3. Mean Fst values across Pairwise set of the complete data in MAF bins. * - In each MAF bin 78 Fst values was averaged. Statistical estimates were obtained with t-test. ** - MSE calculation see at materials and methods. Table S4. Estimates of Fst values calculated for H0 distribution. Fst values should be multiplied by 10− 4. Table S5. Standard errors of the Fst – values computed by EIGENSOFT 6.0.1. Standard errors of Fst obtained from complete data are above diagonal and from pruned data are below diagonal. SE values should be multiplied by 10− 4. Table S6. Description of the herds and number of the genotyped cows. * - Country of origin of the sires of the genotyped cows, NL – the Netherlands. Figure S1. Effect of LD - based pruning on the number of SNP in the complete data. [file 12863_2020_848_MOESM1_ESM.zip › Additional material.docx]
