# Supplementary figures and images for: Assessing the power of principal components and wright’s fixation index analyzes applied to reveal the genome-wide genetic differences between herds of Holstein cows
Source: BMC Genet. 2020 Apr 28;21:47. doi: 10.1186/s12863-020-00848-0 (PMC7189535; doi:10.1186/s12863-020-00848-0)

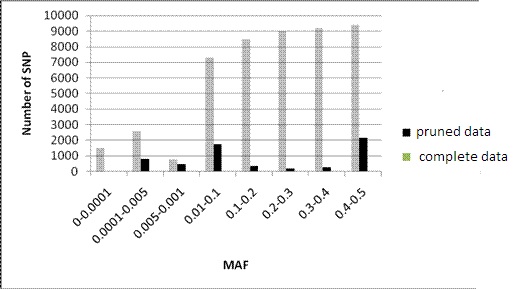

Supplement: Supplementary file 1 — Additional file 1: Table S1. Effect of outliers on estimates of Fst values for complete data. a - Fst values for complete data corrected on the outliers are above the diagonal and Fst values for complete data does not corrected on the outliers are below the diagonal. b - Increased Fst values are in bold and decreased Fst values are in bold Italic. Table S2. Effect of rare alleles with MAF < 0.01 on estimates of Fst values. a - Fst values for complete data after removal of the alleles with MAF < 0.01 are below the diagonal and Fst values for complete data does not corrected on MAF < 0.01 are above the diagonal. b - increased Fst values are in bold and decreased Fst values are in bold Italic. Table S3. Mean Fst values across Pairwise set of the complete data in MAF bins. * - In each MAF bin 78 Fst values was averaged. Statistical estimates were obtained with t-test. ** - MSE calculation see at materials and methods. Table S4. Estimates of Fst values calculated for H0 distribution. Fst values should be multiplied by 10− 4. Table S5. Standard errors of the Fst – values computed by EIGENSOFT 6.0.1. Standard errors of Fst obtained from complete data are above diagonal and from pruned data are below diagonal. SE values should be multiplied by 10− 4. Table S6. Description of the herds and number of the genotyped cows. * - Country of origin of the sires of the genotyped cows, NL – the Netherlands. Figure S1. Effect of LD - based pruning on the number of SNP in the complete data. [file 12863_2020_848_MOESM1_ESM.zip › Figure S1_additiolal file.jpg]
